# Increase of blood-brain barrier leakage is related to cognitive decline in vascular mild cognitive impairment

**DOI:** 10.1186/s12883-021-02189-6

**Published:** 2021-04-15

**Authors:** Man Li, Yue Li, Long Zuo, Wenli Hu, Tao Jiang

**Affiliations:** 1 Radiology Department, Beijing Chao-Yang Hospital, Capital Medical University, No. 8, South Gongti Road, Chaoyang District, Beijing, 100020 P.R. China; 2 Neurology Department, Beijing Chao-Yang Hospital, Capital Medical University, No. 8, South Gongti Road, Chaoyang District, Beijing, P.R. China

**Keywords:** Blood-brain barrier, Vascular mild cognitive impairment, Dynamic contrast-enhanced-magnetic resonance imaging, Cognitive function

## Abstract

**Background:**

Blood-brain barrier (BBB) breakdown, as an early biomarker for vascular mild cognitive impairment (vMCI), has only been validated by a few studies. The aim of this study was to investigate whether compromised BBB integrity is involved in vMCI patients, and detect the relationship between BBB breakdown and cognitive function. BBB leakage in vMCI was explored, and the relationship between BBB leakage and cognitive function was discussed in this study.

**Methods:**

This is a cross-sectional study involving 26 vMCI patients and 21 sex- and age-matched healthy controls. Dynamic contrast-enhanced-magnetic resonance imaging was performed for all participants, to determine BBB leakage. Leakage volume, leakage rate, and fractional blood plasma volume (Vp) in the grey and white matter were evaluated. Neuropsychological tests were used to determine cognitive function. Leakage rate, leakage volume, and Vp in different brain locations, including deep grey matter, cortical grey matter, white matter hyperintensity, and normal-appearing white matter were compared between the two groups.

**Results:**

Multivariable linear regression analyses revealed that in all regions of interest, the leakage rate was significantly higher in vMCI patients relative to controls. Leakage volume in normal-appearing white matter and white matter hyperintensity were significantly higher, while Vp in normal-appearing white matter, deep grey matter, and cortical grey matter were significantly lower in vMCI patients. Moreover, Montreal Cognitive Assessment scores decreased with the increase of leakage rate in white matter hyperintensity.

**Conclusion:**

Increased BBB permeability was detected in vMCI patients and was related to cognitive decline, which suggested that BBB breakdown might be involved in cognitive dysfunction pathogenesis.

**Supplementary Information:**

The online version contains supplementary material available at 10.1186/s12883-021-02189-6.

## Background

Vascular cognitive impairment (VCI) includes subjective cognitive decline, vascular mild cognitive impairment (vMCI), and dementia [1]. Vascular dementia is the second most common subtype of dementia, and comprises about 14.5% of the total number of dementias [2]. VMCI is considered as an early stage of VCI, and it is most prevalent in elderly Chinese [3]. Its pathophysiology remains unclear, but various pathophysiological processes may affect its progress, including blood-brain barrier (BBB) defects, inflammation altered vascular reactivity, hypoperfusion, and innate immune processes [4–6].

It had been studied that BBB breakdown occurs in the early stage of cognitive dysfunction [7]. Neuropathological, cerebrospinal fluid (CSF) biomarker, and non-quantitative neuroimaging studies have revealed the increase of BBB permeability in cognitively declining patients [8–10]. Some studies used dynamic contrast-enhanced-magnetic resonance imaging (DCE-MRI) to quantitatively evaluate BBB permeability in cerebral small vessel disease(cSVD) and stroke [7, 11, 12], showing that subtle BBB leakage was detected. Although increased BBB leakage in white matter was found in patients with VCI using DCE-MRI [13], there were scarce studies focusing on vMCI patients, and understanding of the relationship between cognitive function and BBB leakage was deficient. The aim of this study was to investigate whether BBB permeability change in different brain regions in vMCI patients. In addition, the matter whether the cognitive function was independently associated with BBB leakage change was tentatively examined.

## Methods

### Study participants

This is a cross-sectional study. Participants were recruited through Beijing Chao-Yang Hospital, Capital Medical University from May 2016 to June 2019. Twenty-six vMCI patients were enrolled, along with 21 sex- and age-matched healthy controls. All participants were subjected to neuropsychological evaluation and DCE-MRI to assess BBB permeability. Participants were recruited into the vMCI group if they met the following criteria: (1) complaints of cognitive function, activities of daily living may be normal or mildly impaired, (2) objective cognitive impairment in at least one cognitive domain, (3) MRI findings suggest that cognitive impairment is associated with vascular diseases [14, 15]. The MRI findings include:(1) > two lacunar infarcts outside the brainstem, (2) Single lacunes(> 5 mm)placed strategically in the striatum or the thalamus, (3)confluent deep WMH (Fazekas score = 2 or 3) or irregular periventricular WMH extending into deep white matter (Fazekas score = 3) combined with or without lacunes or microbleeds [14].Sex- and age-matched controls were recruited which presented for physical examination at the medical examination centre of Beijing Chao-Yang Hospital. Exclusion criteria included psychiatric disorders, alcohol/drug abuse, epilepsy, brain tumor, brain trauma, vascular malformation, cardiac and systemic disease, neurodegenerative disease, Parkinson disease, intracranial infection, and contraindications to MRI or intravenous gadolinium.

### Ethics

Ethical clearance for this study was given by the institutional ethics committee of Beijing Chao-Yang Hospital, Capital Medical University. All participants provided signed informed consent before participating in the study.

### Neuropsychological evaluation

All participants underwent a face-to-face neuropsychological test by two trained interviewers within 3 days before or after MRI examination. We calculated cognitive function using Mini-Mental State Examination (MMSE), Montreal Cognitive Assessment (MoCA), Digit Span Test (DST, including forward and backward), Trail-Making Test (TMT, including Part A and Part B), Symbol Digital Modalities Test (SDMT), Stroop color word Test (SCWT, including Part A, B and C), Clock Drawing Test (CDT, 4-point scale), and verbal fluency test (VFT). Overall cognitive function was assessed by MMSE and MoCA. Other cognitive domains included memory (delayed racall test in MoCA, DST), information processing speed (TMT-A), attention (SDMT), executive function (TMT B-A, SCWT C-B), spatial function (CDT), and language function (VFT) [14, 16].

### Imaging protocol and analysis

MRI examinations were executed using a Siemens 3 T MR system(Siemens, Prisma, Munich, Germany), with a 64-channel array head coil. Structural MRI was first performed(See Supplementary Table 1, Additional file 1). DCE-MRI was then performed. The T1 DCE protocol was a fast sequence (repetition time/echo time [TR]/[TE]5.08/1.8 ms, flip angle 15°, slice number = 20 per volume, voxel size 1.2 × 1.2 × 3 mm^3^, field of view [FOV]23 × 23 cm^2^), this sequence included 60 volumes of continuous acquisition. Gadolinium (1.0 mmol/ml, at an injection volume of 0.1 mmol/kg body weight and an injection speed of 2.5 ml/s) was injected using a high-pressure injector after the acquisition of 4 volumes T1WI, flushing with 20 mL saline. Before dynamic imaging, baseline T1 mapping was performed using two flip angles (3°, 15°). T1 mapping [17] was done to convert the contrast-enhanced signal strength into concentration in the tissue.

**Fig. 1:**
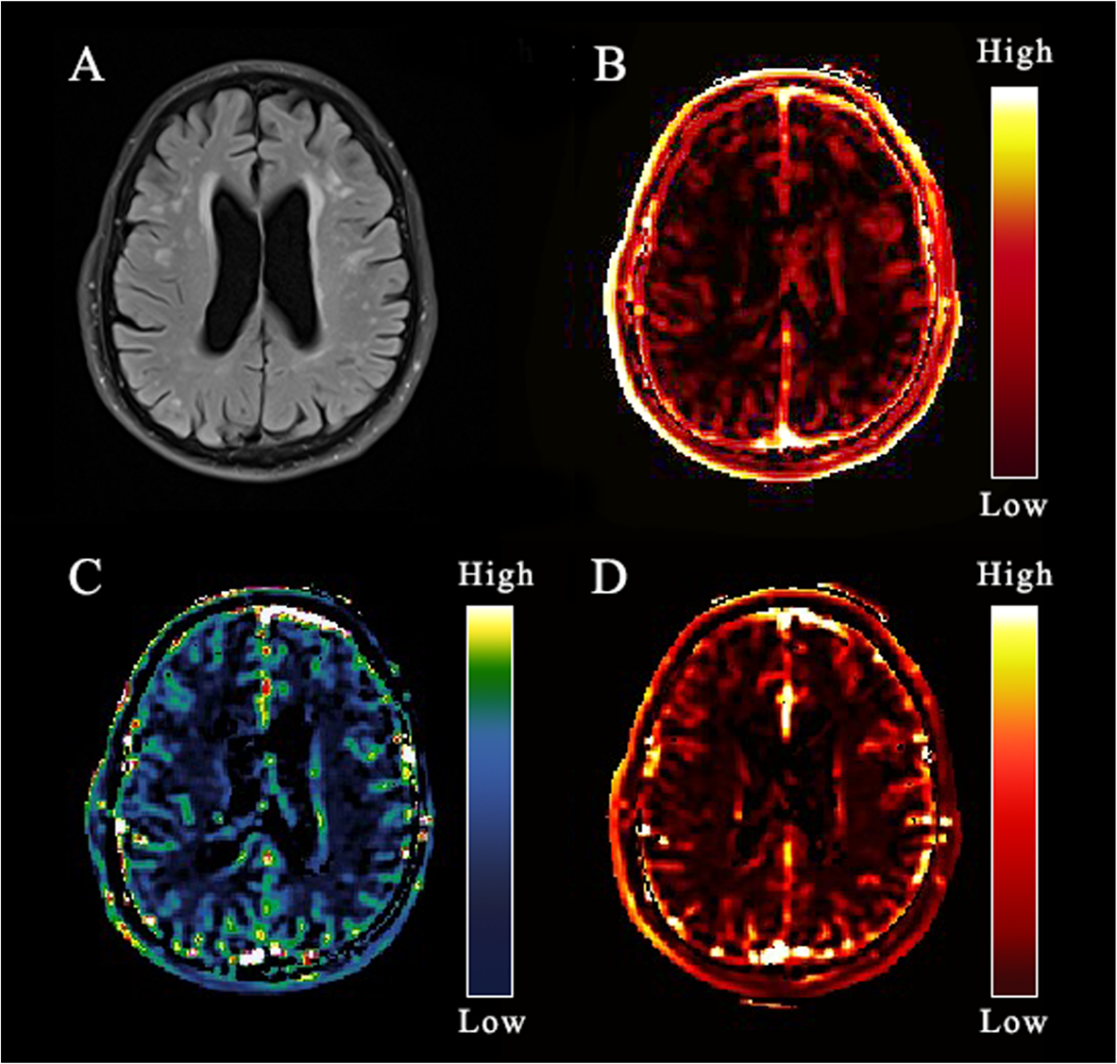
An example map. **a** Axial fluid-attenuated inversion recovery (FLAIR) image of a79-year-old man; **b** BBB leak (K_trans_) map; **c** Area under the leakage curve (AUC) map; **d** Fractional blood plasma volume (V_p_) map

**Fig. 2:**
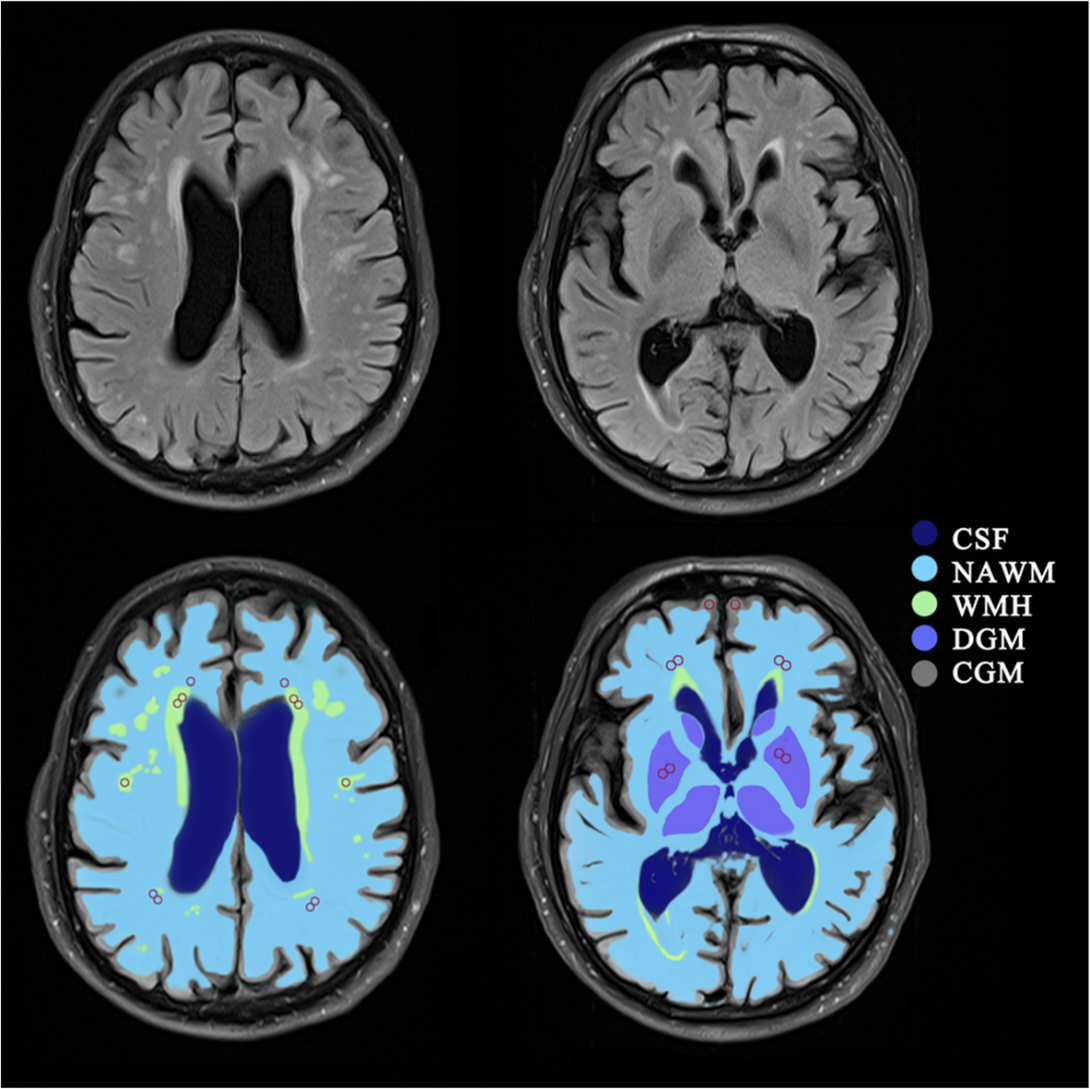
Regions of interest (ROIs). Example of the template for sampling ROIs (red circles) in normal-appearing white matter (NAWM), white matter hyperintensities (WMH), cortical gray matter (CGM), and deep gray matter (DGM). CSF, cerebrospinal fluid

DCE-MRI post-processing was carried out using Nordic ICE (Nordic Neuro Lab). Vascular input function was gained from the superior sagittal sinus [18]. Patlak model was applied to assess the subtle BBB leakage [19]. This approach obtained 3 parameters: area under the leakage curve (AUC), BBB leakage rate (K_trans_), and V_p_ (Fig. 1). Regions of interest (ROIs) were manually outlined according to Axial T2 FLAIR sequence. ROIs included 4 areas: deep grey matter (DGM), cortical grey matter (CGM), white matter hyperintensity (WMH), and normal-appearing white matter (NAWM) (Fig. 2). The ROIs of control subjects were similar in size and location to those of patients. ROIs were 5 mm^2^ in area. Medial temporal lobe atrophy (MTA) scale was used to measure the hippocampal atrophy on coronal T1-weighted MRI [20].

### Statistical analysis

Independent sample *t*-test, Fisher’s exact test, Mann-Whitney U test, and χ^2^ test were used to compare the characteristics of participants and cognitive function in the vMCI vs control group. BBB leakage and V_p_ of all ROIs between the two groups were compared using univariable linear regression analysis, multivariable linear regression analysis was performed to correct sex, age, and vascular risk factors. The relationship between cognitive function and BBB leakage was assessed using Spearman correlation analysis. Next, associations of BBB leakage with cognitive function were investigated using univariable linear regression analyses; then, multivariable linear regression analyses were performed to correct vascular risk factors, sex, age, and education years. *P* < 0.05 indicated statistical significance.

## Results

### Participants characteristics

Fifty subjects participated in this study, of which, three were excluded due to MRI contraindication, image artifacts, or brain tumor. Of the remaining 47 participants, 26 had vMCI, and 21 were sex- and age-matched controls. Analysis of baseline characteristics revealed significant differences in hypertension between the two groups (*P* < 0.001; Table 1).

**Table 1:** Clinical characteristics of participants at baseline

	vMCI group *N* = 26	Control *N* = 21	*P*
Male, N (%)	12(46.2%)	8(38.1%)	0.583
Age, years	71.04 ± 8.99	66.67 ± 9.19	0.108
Education, years	11.58 ± 3.18	11.62 ± 3. 49	0.966
Vascular risk factors			
BMI, kg/m^2^	26.02 ± 4.21	25.72 ± 2.42	0.774
Hypertension, N(%)	26(100.0%)	11(52.4%)	<0.001
Current Smoking, N(%)	4(15.4%)	3(14.3%)	0.623
T2DM, N(%)	4(15.4%)	3(14.3%)	0.623
Hyperlipidemia, N(%)	20(76.9%)	11(52.4%)	0.081
MTA scale			
Right(median (range))	0(0,1)	0(0,1)	0.085
Left(median (range))	0(0,1)	0(0,1)	0.085
MoCA	23.04 ± 2.32	28.00 ± 1.22	<0.001
MMSE	26.31 ± 1.12	29.24 ± 0.70	<0.001

Data are presented as mean ± SD, or counts (%)

*Abbreviations*: *N* number of persons, *vMCI* vascular mild cognitive impairment, *BMI* body mass index, *T2DM* type 2 diabetes mellitus, *MTA* medial temporal lobe atrophy, *MoCA* Montreal Cognitive Assessment, *MMSE* mini-mental state examination

### Cognitive function

Analysis of cognitive functions revealed significant differences in MoCA, MMSE, information processing speed, attention executive function, spatial function, and language function between the two groups. The specific data are shown in Table 2.

**Table 2:** Cognitive function in different domains of participants

	vMCI group *N* = 26	Control *N* = 21	*P*
Overall cognitive function			
MMSE	26.31 ± 1.12	29.24 ± 0.70	<0.001
MoCA	23.04 ± 2.32	28.00 ± 1.22	<0.001
Memory			
Delayed recall	4.62 ± 0.50	4.86 ± 0.36	0.059
DST-forward	7.85 ± 0.78	8.33 ± 0.97	0.063
DST-backward	3.77 ± 0.65	4.10 ± 0.70	0.106
Information processing speed			
TMT-A	59.23 ± 10.88	39.81 ± 9.11	<0.001
Attention			
SDMT	31.81 ± 4.45	39.57 ± 6.52	<0.001
Executive function			
TMT B-A	85.96 ± 31.97	56.71 ± 19.54	0.001
SCWT C-B	68.31 ± 24.81	49.81 ± 21.02	0.009
Spatial function			
Clock Drawing Test	3.31 ± 0.55	3.81 ± 0.40	0.001
Language function			
VFT	42.38 ± 10.09	51.43 ± 7.21	0.001

Data are presented as mean ± SD, or counts (%)

*Abbreviations*: *N* number of persons, *vMCI* mild vascular cognitive impairment, *MMSE* Mini-Mental State Examination, *MoCA* Montreal Cognitive Assessment, *DST* digit span test, *SDMT* symbol digital modalities test, *SCWT* stroop color word test, *CDT* clock drawing test, *VFT* verbal fluency test

### Correlation between BBB leakage and vMCI

Quantitative analysis and comparison results between the two groups are shown in Table 3. Univariable and multivariable (corrected for vascular risk factors, sex, and age) linear regression analyses showed that participants in vMCI group had higher BBB leakage rate in all ROIs and higher BBB leakage volume in NAWM and WMH (Table 3).

**Table 3:** Leakage rate, area under the leakage curve, and fractional blood plasma volume in patients with vascular mild cognitive impairment (vMCI) and controls

	vMCI group	Control	Univariable ^a^		Multivariable ^b^	
			β *P*		β *P*	
**NAWM**						
K_trans_ (10^− 4^ min^− 1^)	0.31 ± 0.14	0.20 ± 0.17	− 0.109	0.016	− 0.109	0.016
AUC	4.41 ± 1.04	3.52 ± 1.20	−0.886	0.008	− 0.888	0.006
Vp (10^–2)^	4.76 ± 1.97	7.51 ± 2.33	2.751	<0.001	2.751	<0.001
**WMH**						
K_trans_ (10^−4^ min^−1^)	0.56 ± 0.24	0.29 ± 0.18	−0.275	<0.001	−0.275	<0.001
AUC	6.02 ± 1.77	4.13 ± 1.25	−1.896	<0.001	−1.896	<0.001
Vp (10^−2^)	8.43 ± 4.39	10.49 ± 5.04	2.058	0.129	0.218	0.115
**CGM**						
K_trans_ (10^−4^ min^−1^)	1.57 ± 0.70	0.93 ± 0.56	−0.642	0.001	−0.642	0.001
AUC	17.90 ± 4.37	16.10 ± 5.76	−1.798	0.217	0.453	0.800
Vp (10^−2^)	20.92 ± 7.97	30.12 ± 9.73	9.192	0.001	9.170	<0.001
**DGM**						
K_trans_ (10^−4^ min^−1^)	0.99 ± 0.50	0.54 ± 0.35	−0.442	0.001	−0.442	0.001
AUC	11.44 ± 2.11	10.24 ± 3.43	−1.200	0.123	0.122	0.429
Vp (10^−2^)	15.36 ± 5.51	19.79 ± 7.10	4.434	0.017	4.445	0.009

Data are presented as mean ± SD

*Abbreviations*: *NAWM* normal-appearing white matter, *WMH* white matter hyperintensities, *CGM* cortex grey matter, *DGM* deep grey matter, *K*_*trans*_ leakage rate, *AUC* area under the leakage curve, *V*_*p*_ fractional blood plasma volume

### Correlation between fractional blood plasma volume and vMCI

Univariable and multivariable (corrected for vascular risk factors, sex, and age) linear regression analyses found that the vMCI group had lower Vp in DGM, CGM, and NAWM (Table 3).

### Correlation between BBB leakage and cognitive function

Spearman correlation analysis showed that BBB leakage rate and leakage volume was negatively correlated with MoCA in all ROIs. Multivariable linear regression analysis revealed that the MoCA scores decreased with the increase of leakage rate in WMH to correct sex, age, vascular risk factors, and education years (Table 4).

**Table 4:** Association of leakage rate, area under the leakage curve, and fractional blood plasma volume with MoCA

	Spearman correlation		Univariable ^a^		Multivariable ^b^	
	r	*P*	β	*P*	β	*P*
**NAWM**						
K_trans_ (10^−4^ min^−1^)	−0.384	0.008	−6.260	0.026	−4.718	0.080
AUC	−0.316	0.030	−0.719	0.063	−0.246	0.590
V_p_ (10^–2)^	0.425	0.003	0.463	0.009	0.439	0.011
**WMH**						
K_trans_ (10^−4^ min^−1^)	−0.540	<0.001	−6.205	<0.001	−4.363	0.010
AUC	−0.551	<0.001	−0.780	0.002	−0.511	0.063
V_p_ (10^–2)^	0.122	0.412	0.048	0.631	−0.003	0.977
**CGM**						
K_trans_ (10^−4^ min^−1^)	−0.425	0.003	−1.214	0.061	−0.571	0.358
AUC	−0.264	0.073	−0.140	0.115	0.011	0.913
V_p_ (10^–2)^	0.443	0.002	0.129	0.003	0.132	0.004
**DGM**						
K_trans_ (10^−4^ min^−1^)	−0.437	0.002	−2.322	0.011	−1.346	0.132
AUC	−0.288	0.049	−0.317	0.054	0.006	0.971
V_p_ (10^–2)^	0.267	0.070	0.085	0.237	0.056	0.449

*Abbreviations*: *MoCA* Montreal Cognitive Assessment, *NAWM* normal-appearing white matter, *WMH* white matter hyperintensities, *CGM* cortex grey matter, *DGM* deep grey matter, *K*_*trans*_ leakage rate, *AUC* area under the leakage curve, *V*_*p*_ fractional blood plasma volume

^a^Univariable linear regression analysis with MoCA as dependent variable, and K_trans_, AUC, and V_p_ respectively, as independent variable

^b^Multivariable linear regression analysis with MoCA as dependent variable, and K_trans_, AUC, V_p_, age, sex, vascular risk factors, and education years as independent variables

β, Unstandardized regression coefficient

## Discussion

In this study, it was found that BBB leakage rate rose in WMH, NAWM, CGM, and DGM in vMCI patients compared with the controls. Additionally, leakage volume in vMCI patients also increased in NAWM and WMH relative to controls. MoCA was negatively correlated with the leakage rate in WMH after correcting sex, age, vascular risk factors, and education years. These results indicated that BBB leakage was elevated widespread in brain parenchyma in vMCI patients and was independently related to cognitive decline.

DCE-MRI, as the most reliable noninvasive quantitative neuroimaging method to quantify BBB leakage in vivo [21],has been used to show BBB breakdown in mild cognitive impairment patients in some studies, but only a few studies focused simply on vascular factor. One study paid attention to the white matter area and found evaluated BBB permeability in white matter in VCI using DCE-MRI [13], but failed to quantify BBB permeability in grey matter which was also involved in VCI [22, 23]. A recent study found the increase of BBB leakage volume in CGM, WMH, and NAWM in vMCI and recent small subcortical infarct patients. In contrast, no differences were found in leakage rate [12], which was inconsistent with the results of this study. The disparity may be caused by different participant selection standard that only patients with vMCI diagnosed according to both cognitive function and MRI findings were included, as well as different image acquisition and post-processing methods.

It was found that BBB leakage rate increased widespread in the brain in vMCI patients and cognitive function decreased with the increase of BBB permeability, which coincided with previous research hypotheses, and may be caused by the neurovascular unit (NVU) disruption [9, 24]. Endothelial cells are the most important NVU component, and their dysfunction usually occurs first [25]. Tight junctions (TJs) are located between endothelial cells, and their disruption allows paracellular diffusion of water-soluble molecules from the blood into brain [26], upsetting brain environment homeostasis and impairing BBB function in vMCI patients [27, 28]. These changes are widely distributed in the brain tissue, rather than limited. A recent study found that BBB damage was an early initiating factor of cognitive dysfunction, which was independent of Aβ and tau protein [7]. Alterations in cerebral microvascular structure and neurovascular coupling dysfunction can lead to micro-vascular thrombosis and BBB dysfunction [29, 30]. This process may trigger a series of biochemical events, including microglial and astroglial activation, deposition of blood-borne proteins in the brain, release of pro-inflammatory neurotoxic cytokines, edema, and hemorrhage, eventually leading to neuronal degeneration and cognitive decline [31–33].

VMCI patients had impaired cognitive domains in information processing speed, attention executive function, spatial function, and language function rather than memory. This is in agreement with previous study [34]. And BBB dysfunctions was only correlated with MOCA rather than MMSE. A previous study exhibited that the MoCA was more sensitive than MMSE in the assessment of vMCI, and it had preferential sensitivity to early screening of VCI and evaluation of global cognitive function [35]. In addition, the MOCA only decreased with the increase of leakage rate in WMH, which was consistent with the result of a previous study [12]. A possible reason was that BBB breakdown may result in deposition of harmful substances. It has been found that WMH can mediates iron deposition, thus leading to cognitive impairment [36].

The study also revealed that vMCI patients displayed lower fractional blood plasma volume in CGM, DGM, and NAWM compared with control participants, indicating cerebral vascular hypoperfusion throughout the brain (including white and grey matter) in vMCI patients, which was consistent with the result of a previous study using DCE-MRI in cSVD [11]. It may be caused by structural abnormalities of blood vessels, including narrowing of the lumen and thickening of the wall due to hyaline arteriolosclerosis [37]. Moreover, reduced vascular density and cerebral vascular reactivity would also lead to hypoperfusion [38, 39]. Microvascular endothelial cells can release vasoactive substances to modulate vessel tone and regulate other functional non-vascular cells [40], while pericytes degeneration changes energy matrix transfer to neurons, reducing of cerebral blood flow (CBF) response in brain activation [41]. Changes of these cerebral vessels and local inflammation induced by ischemia may aggravate white matter damage and cognitive dysfunction [38, 42].

However, WMH was not involved, which was consistent with the results of previous studies that CBF was not associated with total WMH volume at baseline [43]. As speculated, the compensatory effect of capillaries may lead to increased local perfusion in WMH. A longitudinal study found that white matter area with low CBF progressed to new WMH during follow-up [44], suggesting that white matter hypoperfusion may contribute to disease progression.

In addition, vMCI is different from the non-amnestic mild cognitive impairment(MCI). MCI is divided into amnestic MCI and non-amnestic MCI with the subtypes of single or multiple domain impaired classification. Both multidomain non-amnestic MCI and multidomain amnestic MCI may be vMCI which is identified based on the evidence of presumed vascular etiology(See Supplementary Figure 1, Additional file 2).

Although this study was limited by its small sample size, significant differences were revealed, indicating the sensibility of the method. It was also limited by the lack of neuropathological biomarkers for indicating increased BBB permeability. However, the results may be confounded by the differences in transport patterns between gadolinium contrast agents and neurobiological markers. Furthermore, the scan time was a bit short, but motion artifacts were reduced, while image signal-to-noise ratio was improved for the application of the 64-channel coil, and significant differences were obtained after multiple measurements, indicating that the method was feasible. Moreover, it is unsure whether the control group will have cognitive impairment in the future, and the control group will be follow up in further study. Finally, no causal relationship between BBB damage and cognitive function was established in this study, which should be further investigated through longitudinal studies.

## Conclusions

The current study found that BBB leakage was higher in both grey matter and white matter in vMCI patients, and cognitive function decreased with the increase of BBB leakage in WMH, which indicated that generalized BBB disruption in vMCI patients could contribute to the pathophysiological mechanisms of impaired cognitive function.

## Supplementary Information

**Additional file 1: Supplementary Table 1.** Structural MRI scanning parameters.

**Additional file 2: Supplementary Figure 1.** How to distinguish different subtypes of MCI and how they can be paired with possible etiologies. This is referenced from Petersen RC: Mild Cognitive Impairment. *Continuum (Minneap Minn)* 2016, **22**(2 Dementia):404–418.

## Data Availability

All data derived from this study are available upon reasonable request.

## Abbreviations

AUC: Area under the leakage curve
BBB: Blood-brain barrier
CGM: Cortical gray matter
CSF: Cerebrospinal fluid
cSVD: Cerebral small vessel disease
CBF: Cerebral blood flow
CDT: Clock drawing test
DCE-MRI: Dynamic contrast-enhanced-magnetic resonance imaging
DGM: Deep gray matter
DST: Digit span test
FOV: Field of view
MCI: Mild cognitive impairment
MoCA: Montreal Cognitive Assessment
MTA: Medial temporal lobe atrophy
NAWM: Normal-appearing white matter
NVU: Neurovascular unit
ROIs: Regions of interest
SDMT: Symbol digital modalities test
SCWT: Stroop color word test
TMT: Trail-making test
TR: Repetition time
TE: Echo time
TJs: Tight junctions
vMCI: Vascular mild cognitive impairment
Vp: Fractional blood plasma volume
VCI: Vascular cognitive impairment
VFT: Verbal fluency test
WMH: White matter hyperintensities
