# Supplementary material for: Increase of blood-brain barrier leakage is related to cognitive decline in vascular mild cognitive impairment
Source: BMC Neurol. 2021 Apr 15;21:159. doi: 10.1186/s12883-021-02189-6 (PMC8048027; doi:10.1186/s12883-021-02189-6)
Supplement: Supplementary file 1 — Additional file 1: Supplementary Table 1. Structural MRI scanning parameters. [file 12883_2021_2189_MOESM1_ESM.doc]

| SEQUENCE | FLAIR | T2WI | T1WI | DCE-MRI | SWI |
| --- | --- | --- | --- | --- | --- |
| ORIENTATION | AX | AX | AX | AX | AX |
| TR | 9000ms | 5000ms | 2000ms | 5.08ms | 28ms |
| TE | 81ms | 94ms | 9ms | 1.8ms | 20ms |
| FOV | 24 | 24 | 24 | 24 | 24 |
| SLICE THICK | 5mm | 5mm | 5mm | 3mm | 1.2mm |
| SLICE GAP | 6.5mm | 6.5mm | 6.5mm | 0mm | 0mm |
| MATRIX | 320×256 | 320×320 | 320×217 | 192×154 | 384×269 |
| FLIP ANGLE |  |  |  | 15° |  |
| DCE-INTERVAL |  |  |  | 3.8sec |  |

**Supplementary Table 1** Structural MRI scanning parameters

**Abbreviations:** FLAIR, fluid attenuated inversion recovery;T2WI, T2-weighted imaging；T1WI, T1-weighted imaging; DCE-MRI, Dynamic contrast-enhanced magnetic resonance imaging; SWI, Susceptibility weighted imaging; AX: axial; TR: repetition time; TE: echo time; FOV: field of view.
