# Supplementary material for: Increase of blood-brain barrier leakage is related to cognitive decline in vascular mild cognitive impairment
Source: BMC Neurol. 2021 Apr 15;21:159. doi: 10.1186/s12883-021-02189-6 (PMC8048027; doi:10.1186/s12883-021-02189-6)
Supplement: Supplementary file 2 — Additional file 2: Supplementary Figure 1. How to distinguish different subtypes of MCI and how they can be paired with possible etiologies. This is referenced from Petersen RC: Mild Cognitive Impairment. Continuum (Minneap Minn) 2016, 22(2 Dementia):404–418. [file 12883_2021_2189_MOESM2_ESM.docx]

**Supplementary Figure 1** How to distinguish different subtypes of MCI and how they can be paired with possible etiologies.

AD = Alzheimer disease; DLB = dementia with Lewy bodies; FTD = frontotemporal dementia; MCI = mild cognitive impairment.

This is referenced from Petersen RC: **Mild Cognitive Impairment**. *Continuum (Minneap Minn)* 2016, **22**(2 Dementia):404-418.

No

Yes

Yes

No

Yes

No

Yes

No

No

Yes

Mild cognitive impairment

Memory impairment?

Amnestic MCI single domain

Amnestic MCI multiple domain

Nonamnestic MCI

single domain

Nonamnestic MCI

multiple domain

FTD or DLB

AD or depression

Evidence for predominantly vascular etiology of cognitive impairment

Vascular MCI

DLB

Amnestic MCI

Nonamnestic MCI

Memory impaired only?

Single nonmemory cognitive domain impaired?

Evidence for predominantly vascular etiology of cognitive impairment

Evidence for predominantly vascular etiology of cognitive impairment

Single nonmemory cognitive domain impaired?

Memory impaired only?

Nonamnestic MCI

Amnestic MCI

DLB

Vascular MCI

Evidence for predominantly vascular etiology of cognitive impairment

FTD or DLB

Nonamnestic MCI

multiple domain

Nonamnestic MCI

single domain

Amnestic MCI multiple domain

Memory impairment?

Mild cognitive impairment

Yes

No

No

Yes

No

Yes
